# The Differential Diagnosis of Neuralgia

**Published:** 1889-04

**Authors:** Daniel R. Brower

**Affiliations:** Professor of Diseases of Nervous System, Woman’s Medical College, Chicago; 597 West Jackson Street


### THE DIFFERENTIAL DIAGNOSIS OF NEURALGIA.

BY DANIEL R. BROWER, M.D.

PROFESSOR OF DISEASES OF NERVOUS SYSTEM, WOMAN'S MEDICAL COLLEGE, CHICAGO.

We define neuralgia as a nervous disorder characterized by pain, and unconnected, so far as we know, with disease of structure.

There are great difficulties in the way of the diagnosis, growing largely out of the fact that its principal symptom is subjective, and may therefore present the most surprising varieties, due to idiosyncrasies. The essential characteristic symptom, however, is pain, which is limited to a definite trunk or branch of the nerve and its ramifications, and is usually unilateral. This pain is paroxysmal, either intermitting, or, at least, remitting in its character, and it is intensely acute and subject to a great variety of characteristics. There are certain points in the course of the nerve, or in its area of distribution, that are sensitive to pressure (points douloureux); and this pain has certain concomitant sensory symptoms, irradiation to other sensory nerves, and hyperæsthesia or anæsthesia of the skin in the region supplied by the affected nerve. Along with the pain there may be concomitant motor phenomena of a convulsive or paralytic type. There are certain vaso-motor disturbances, such as a diffuse or more or less redness or pallor of the skin; a sensation of coldness, and certain secretory phenomena, such as in the case of neuralgia of the fifth nerve; abundant lachrymation, nasal catarrh, and salivation, and in all cases there may be an increase in the urinary and cutaneous secretions, and certain trophic phenomena, such as change in the color of the hair, hypertrophy and thickening of various tissues, and also atrophy and emaciation, with skin affections, such as herpes zoster, urticaria, etc. The neuralgia is unaccompanied by any inflammatory or local symptom, or any general disturbance of the health, commensurate with the amount of pain endured.

*Neuralgia of the fifth nerve* may usually be diagnosticated without much difficulty by the character of the pain, its intensity, its lancinating and paroxysmal character, by the painful points being well defined at the supra-orbital foramen, the infra-orbital foramen, and the mental foramen. It may be mistaken for tooth-ache; careful examination of the teeth usually differentiates this. It may be mistaken for inflammation of the periosteum of the facial bones, or the membrane lining the antrum and frontal sinuses. The kind of pain, its seat, and the degree of sensibility to pressure usually differentiate this.

The diagnosis of the seat of neuralgia, as to whether it is peripheral or central, can only be made in a very few cases. We infer its peripheral seat if we find some evident peripheral cause from the possibility of cutting short the attack by remedies applied to the periphery, from the presence of painful points during the interval between the paroxysms, and the limitation of the pain to a single branch of the nerve. A point in favor of its central origin is the absence of painful points and the interval between the attacks, the disturbance of other cerebral nerves, associated with marked mental symptoms.

The diagnosis of *cervico-occipital neuralgia* is usually easily made. It is only liable to be confounded with muscular rheumatism, and its differentiation from this should not be difficult.

*Cervico-brachial neuralgia* requires special care in its diagnosis since the arm may be the seat of other painful affections, such as muscular and articular rheumatism, diseases of the bone, etc. Differentiation here is aided by the "points douloureux." They are the axillary, corresponding to the brachial plexus, the scapular, near the inferior angle of the scapula, the acromical, in the angle between this process and the clavicle, the median cephalic in the bend of the elbow, the ulnar corresponding to the most superficial portion of the ulnar nerve at the back of the elbow joint, and the radial at the point where the radial nerve becomes superficial. These puncta dolorosa would be absent in the affections for which the disease may be mistaken.

*Dorso-intercostal neuralgia* is most frequently confounded with rheumatism of the thoracic muscles. The painful points are (1) close to the vertebral column corresponding to the point where the nerve emerges from the inter-vertebral foramen; (2) where the nerve emerges from beneath the skin at a point about the middle of the entire course of the nerve; and (3) where the nerve pierces the muscles close to the sternum.

A consideration of these painful points, in the absence of any special pain in the muscles, or any aggravation on making movements, will usually differentiate them.

*Intercostal neuralgia* may be mistaken for *angina pectoris*, but the intense fear, great anxiety, sense of suffocation, and disorder of the circulation attending the latter, will usually make differentiation easy.

*Lumbo-abdominal neuralgia.* The diagnosis is not unattended with difficulty. It may be confounded with myalgia, especially lumbago. In lumbago, however, the pain is usually circumscribed, does not radiate, is aggravated by stretching of the body and disappears with rest. The differentiation of this form of neuralgia from hip and knee-joint disease requires special care and attention.

The diagnosis of *sciatica* is often surrounded by considerable difficulty. The principal painful points are those which correspond to the sacral foramina, the fibular point at the head of the fibula, and the external and internal malleolar points. It may be mistaken for myalgia, differentiation being made by the diffuse character of the myalgic pain and by its being increased by definite movements. Sciatica may be mistaken for hip-joint disease, and the greatest care is necessary to avoid error here. The absence or presence of pain when the head of the femur is pressed against the acetabulum; the age and state of the general health; the elongation or shortening of the leg; the shape of the lower part of the back; attention to the painful points of neuralgia; the paroxysmal character of the pain, and its mode of distribution; the presence or absence of fever and inflammation, and the mode of carrying up the leg, are all to be considered in making the diagnosis. The sharp pains of locomotor ataxia are more frequently felt in the legs than in any other part of the body, and are often mistaken for neuralgia. Differentiation here is made by considering their changing seat and momentary duration, by their not following the nerve trunks; by the absence of the painful points, and by their frequently being so localized that the point of the finger will cover the seat of the pain; by the condition of the pupillary and patellar-tendon reflexes, and by the incöordination of muscular movements.

597 West Jackson Street.

